# Tea Tree Oil Prevents Mastitis-Associated Inflammation in Lipopolysaccharide-Stimulated Bovine Mammary Epithelial Cells

**DOI:** 10.3389/fvets.2020.00496

**Published:** 2020-08-07

**Authors:** Zhi Chen, Yi Zhang, Jingpeng Zhou, Lu Lu, Xiaolong Wang, Yusheng Liang, Juan J. Loor, Deming Gou, Huifen Xu, Zhangping Yang

**Affiliations:** ^1^College of Animal Science and Technology, Yangzhou University, Yangzhou, China; ^2^Joint International Research Laboratory of Agriculture & Agri-Product Safety, Ministry of Education, Yangzhou University, Yangzhou, China; ^3^Mammalian Nutrition Physiology Genomics, Division of Nutritional Sciences, Department of Animal Sciences, University of Illinois, Urbana, IL, United States; ^4^College of Life Sciences, Shenzhen University, Shenzhen, Guangzhou, China; ^5^College of Animal Science and Veterinary Medicine, Henan Agricultural University, Zhengzhou, Henan, China

**Keywords:** TTO, BMEC, LPS, mastitis, transcriptome sequencing

## Abstract

The main purpose of this study was to explore the effect of tea tree oil (TTO) on lipopolysaccharide (LPS)-induced mastitis model using isolated bovine mammary epithelial cells (BMEC). This mastitis model was used to determine cellular responses to TTO and LPS on cellular cytotoxicity, mRNA abundance and cytokine production. High-throughput sequencing was used to select candidate genes, followed by functional evaluation of those genes. In the first experiment, LPS at a concentration of 200 μg/mL reduced cell proliferation, induced apoptosis and upregulated protein concentrations of tumor necrosis factor-α (TNF-α), interleukin 6 (IL-6), and signal transducer and activator of transcription 1 (STAT1). Addition of TTO led to reduced cellular apoptosis along with downregulated protein concentrations of nuclear factor kappa B, mitogen-activated protein kinase 4 (MAPK4) and caspase-3. In the second experiment, BMEC challenged with LPS had a total of 1,270 differentially expressed genes of which 787 were upregulated and 483 were downregulated. Differentially expressed genes included *TNF*-α, *IL6, STAT1*, and *MAPK4*. Overall, results showed that TTO (at least *in vitro*) has a protective effect against LPS-induced mastitis. Further *in vivo* research should be performed to determine strategies for using TTO for prevention and treatment of mastitis and improvement of milk quality.

## Introduction

Lipopolysaccharide (LPS) is one of the main components of the cell wall of gram-negative bacteria including *Escherichia coli* (*E. coli*) and other mastitis-inducing pathogenic bacteria such as *Staphylococcus aureus, Streptococcus agalactis*, and *Streptococcus lactis* (1). In dairy cows, mastitis caused by *E. coli* results in increased concentrations of acute-phase proteins in milk (2, 3), and can be treated with antibiotics (4). However, with increasing concerns about drug resistance it has become imperative to prevent usage of antibiotics and develop alternatives and treat cow mastitis using alternative therapies.

Tea tree oil (TTO; terpinen-4-ol type), also known as *M. alternifolia* oil, is an essential oil from several plants of Melaleuca, of which the main one is *M. alternifolia* (5). TTO is widely used in many over-the-counter health products and cosmetics. With the vigorous development of natural and alternative medicinals, an increasing number of people are using products containing TTO (6). TTO has a broad antibacterial spectrum and strong antibacterial activity, which explains its use to treat diseases caused by fungi, bacteria, or viruses (7). Therefore, its potential use as a natural antibacterial agent to replace antibiotics as a component of mastitis therapy is of interest.

With the development of sequencing and histochemistry technology, analysis of the complex pathogenesis of mastitis in dairy cows from multiple perspectives can be performed. More importantly, an integrative approach aids in effective biomarkers for timely and accurate prevention (8). Although numerous studies have reported alterations of mRNA abundance in the mammary gland in response to mastitis, the role of gene transcription along with the complex networks and how they respond to therapeutic agents is still unclear. For instance, microRNA expression was first confirmed during mastitis in 2007 (9). Naeem et al. detected changes in 14 miRNA in mammary tissue 12 h after infection with *Streptococcus uberis*. Compared with healthy tissue, expression of miR-15b, miR-16a, miR-21, miR-145, and miR-181a was lower, and only miR-223 was greater in infected mammary tissue. The miR-16a was decreased of some interleukins (IL-6, IL-8, and IL-10). The present study aimed to use transcriptome technology to uncover the response of bovine mammary epithelial cells (BMEC) to LPS as a way to identify key candidate genes that could be target for functional verification. Along with other assays, a combined technological approach can provide precise targets for research and development of effective therapeutic drugs, ultimately achieving positive effects in terms of prevention and treatment (10).

## Materials and Methods

### Ethics Statement

The animal use protocol was approved by the Institutional Animal Care and Use Committee in the College of Animal Science and Technology, Yang Zhou University, Yang Zhou, China.

### Culture of BMEC

Three peak lactation dairy cows were selected for mammary gland biopsy (11). After PBS washing, fat tissue and connective tissue were peeled off. The BMEC were separated by the tissue block method followed purification by differential digestion and cryopreservation after subculturing (11). Cells were cultured in Dulbecco's modified Eagle medium/F12 (DMEM/F12) supplemented with 10% (vol/vol) fetal bovine serum in a humidified incubator at 37°C with 5% CO_2_. Medium was replaced every 48 h. The BMEC were digested with 0.25% trypsin for passaging, and the growth of cells was observed using an inverted microscope (11).

### CCK-8 Detection of Cell Proliferation Activity Induced by LPS

The density of BMEC was adjusted to 1 × 10^4^ in a 96-well plate. After 24 h incubation, the culture medium was discarded. The BMEC were treated with LPS (50, 100, 200, 500, and 1,000 μg/mL). In addition, there was a control (BMEC without LPS) and a blank group (only culture medium without cells). After 4, 8, 12, and 24 h incubation, cell proliferation activity was detected using a CCK-8 kit (Watson Technology Co., Ltd., Beijing, China) according to the manufacturer's protocols.

### Detection of Apoptosis Rate Induced by LPS via Flow Cytometry

The BMEC were plated in a 6-well plate and incubated for 24 h. Cells were then washed and collected with PBS, and cell concentration adjusted with buffer to 1 × 10^6^/100 μL/test. Then, 5 μL annexin V-FITC and 5 μL PI were added, and cell apoptosis determined in a dark room.

### Effect of TTO on Apoptosis Rate During LPS Challenge via Flow Cytometry

The BMEC were plated in a 6-well plate and cultured for 24 h. LPS and various concentrations of TTO (Yuanye biology Co., Ltd., Shanghai, China) were added to the culture (0.0002, 0.0004, 0.0006, 0.0008, 0.001, 0.002, 0.004, 0.006, 0.008, and 0.01%, vol/vol). Annexin V-FITC and PI were added for detection of apoptosis.

### Abundance of Inflammation- and Apoptosis-Related Proteins via ELISA

After washing with PBS, RIPA buffer was added to the cell lysate. Bovine nuclear factor kappa B (NF-κB), mitogen-activated protein kinase 4 (MAPK4), tumor necrosis factor-α (TNF-α), interleukin 6 (IL-6), signal transducer and activator of transcription 1 (STAT1), and apoptosis-related caspase-3 were determined according to protocols supplied with the ELISA kits (Qiaoshe Co., Shanghai, China).

### Transcriptome Sequencing

#### Library Construction

Total RNA was extracted from BMEC (number of cells is 1 × 10^7^) treated with 200 μg/mL LPS for 12 h. After total RNA was extracted and digested with DNase, eukaryotic mRNA was enriched with oligo (dT) using magnetic beads. A strand of cDNA was synthesized with random hexamers using the interrupted mRNA as template. Double-stranded cDNA was synthesized using the two-stranded synthesis system and purified followed by poly-(A) addition and sequencing. The library was inspected for quality using the Agilent 2100 Bioanalyzer, and eventually sequenced with the Illumina hiseqtm 2500 sequencer (12). The raw data generated by high-throughput sequencing was in FASTQ format. To obtain high-quality reads, we first used NGS QC Toolkit software to conduct quality control and remove joints.

#### Gene Quantification, Differential Gene Screening, Functional Enrichment, and Cluster Analysis

The comparison between clean reads and the reference genome were stored in a binary file (BAM file). Genes were quantified to obtain the FPKM value using cufflinks. When calculating differences in gene expression, we used Htseq-count software to determine the number of gene reads in each sample. The estimate SizeFactors function in the DESeq R package was used to standardize the data, and the nbinomTest function was used to calculate the *P*-value and fold-change values in the difference comparisons (13). The condition used to screen differentially expressed genes was a *P* < 0.05 and multiple differences was more than 2 times. Gene ontology (GO) and Kyoto Encyclopedia of Genes and Genomes (KEGG) enrichment analysis of differentially expressed genes were carried out to determine main biological functions or pathways.

### H&E Staining of Cells

Cell sections were deparaffinized in xylene, and slides hydrated in 95% ethanol for 5 min, 85% ethanol for 5 min; slides were then hydrated in distilled water. Hematoxylin staining was performed for 3 min, and slides rinsed with distilled water for 2 min; 1% hydrochloric acid alcohol was used for 2 s to differentiate the stain. The sections were rinsed with tap water for 15 min followed by 1–2 s of distilled water. Slides were stained with eosin for 30 s. Differentiation was determined according to the color, and 80% ethanol was used to differentiate stains. Slides were further dehydrated with 85% ethanol for 5 min, followed by 95% ethanol for 5 min. Then, the slides were dehydrated with anhydrous ethanol for 10 min. After the run off was transparent, slides were sealed by adding a drop of neutral gum. Observation and photography were performed with a microscope (Dmi4000b inverted fluorescence microscope, Leica, Germany).

### Immunohistochemistry

Sections were dewaxed and hydrated followed by washing in xylene twice for 10 min each. Slides were then incubated with 100, 95, 85, and 75% ethanol for 5–10 min. The sections were soaked in distilled water for 5 min. For antigen retrieval, sections were incubated in citrate buffer (pH 6.0) and heated in a microwave at high heat for 8 min. Cells were then washed with 1 × PBS (pH 7.2~7.6) three times for 3 min each time followed by addition of 3% H_2_O_2_ at room temperature for 10 min to inactivate endogenous peroxidases. Slides were rinsed with 1 × PBS three times, 3 min each. Slides were incubated with primary antibodies (BV20932, Qiaoshe company, Shanghai, China) followed by a secondary antibody (BV30796, Qiaoshe company, Shanghai, China) in a box at 37°C for 1.5 h. Slides were washed 3 times with 1 × PBS for 5 min each. A streptavidin-HRP antibody was incubated with the slides at 37°C for 20 min. Slides were then covered with 100 μL of the previously prepared color developer DAB working solution; the reaction time was monitored under the microscope.

### Statistical Analysis

Statistical analyses were performed by SPSS 19.0 (SPSS Inc., Chicago, USA). The data are presented as mean values ± s.d. from three independent experiments, duplicates. Statistical analysis was conducted using two-tailed unpaired Student's *t*-test or one-way ANOVA with Bonferroni's multiple comparisons test. *P* < 0.05 was considered significant.

## Results

### CCK-8 Detection of Cell Proliferation Activity Induced by LPS

**Figure 1:**
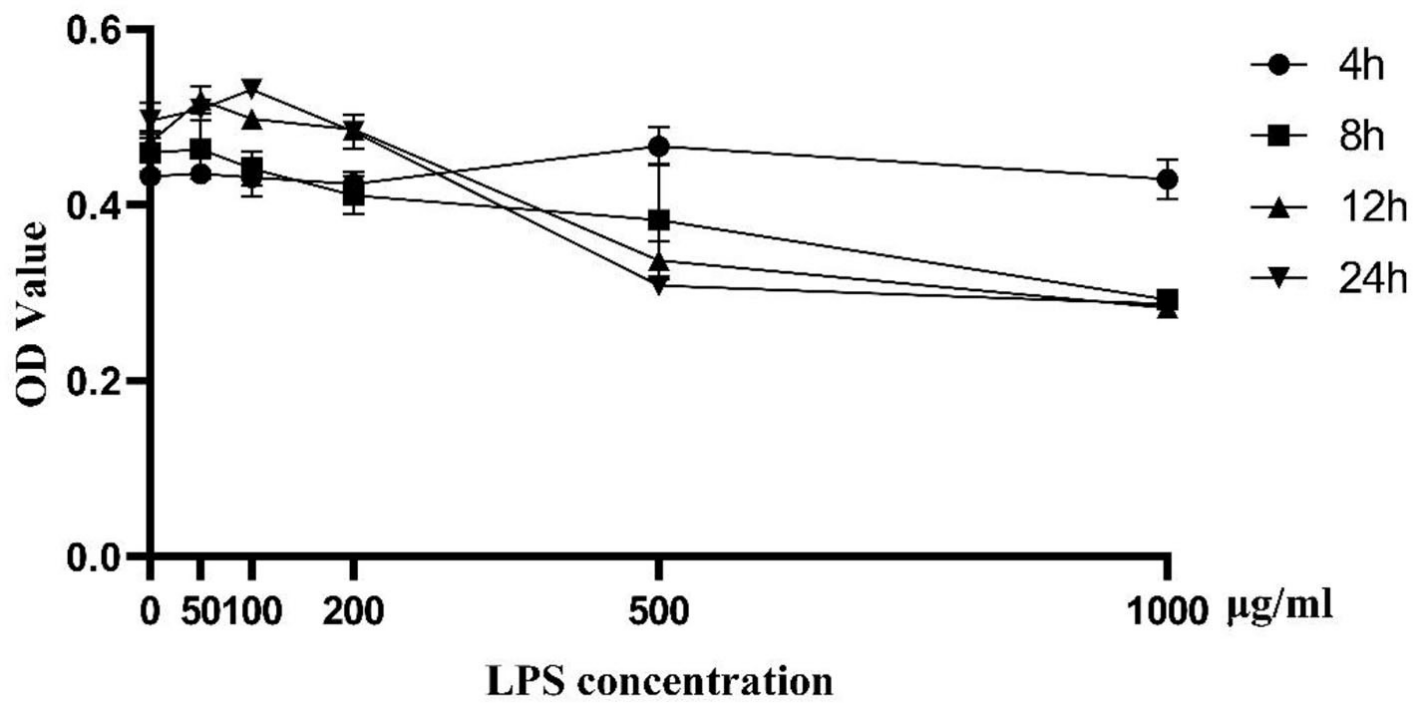
Cell proliferation activity induced by LPS at different concentrations (0, 50, 100, 200, 500, and 1,000 μg/ml) and time points (0, 4, 8, 12, 24 h). Data were presented as means ± s.d. of at least three independent experiments.

As shown in Figure 1, cell proliferation activity of BMEC began to decline to varying degrees with 100 μg/mL LPS treatment for 12 h. As the activity of cells induced by LPS of 500 μg/mL and 1,000 μg/mL was too low, we chose the challenge of LPS concentration at 200 μg/mL for 12 h as the optimal treatment condition for further analysis. Biological repeat is three times, and technical repeat is two times.

### Apoptosis of LPS-Induced BMEC

**Figure 2:**
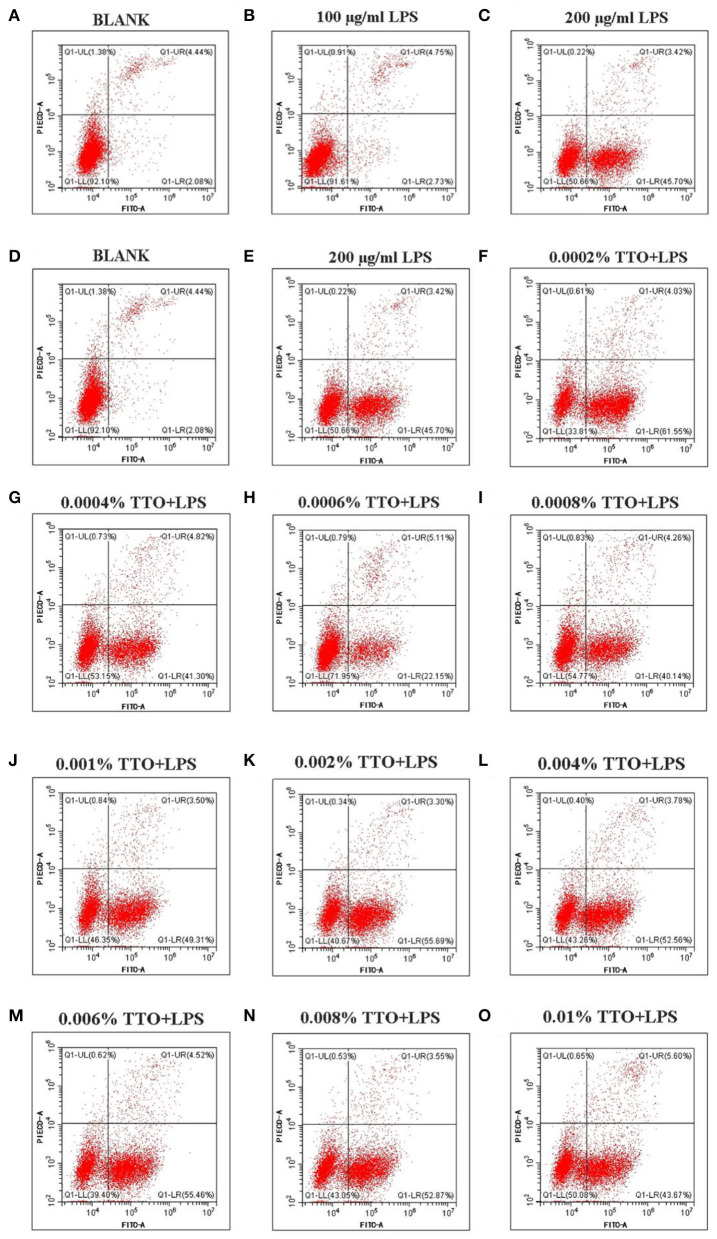
Effect of different concentrations of TTO on apoptosis in BMEC with LPS challenge. **(A)** BLANK; **(B)** 100 μg/ml LPS; **(C)** 200 μg/ml LPS; **(D)** BLANK; **(E)** 200 μg/mL LPS; **(F)** 0.0002% TTO+LPS; **(G)** 0.0004% TTO+LPS; **(H)** 0.0006% TTO+LPS; **(I)** 0.0008% TTO+LPS; **(J)** 0.001% TTO+LPS; **(K)** 0.002% TTO+LPS; **(L)** 0.004% TTO+LPS; **(M)** 0.006% TTO+LPS; **(N)** 0.008% TTO+LPS; **(O)** 0.01% TTO+LPS. Data were presented as means ± s.d. of at least three independent experiments.

Approximately 4.44% (4.44 ± 0.01) early apoptosis and late apoptosis were observed without LPS (Figure 2A). Upon addition of 100 μg/mL LPS, the whole image shifted to the right, and ~7.48% (7.48 ± 0.02) [early apoptosis 2.73 (2.73 ± 0.01) +late apoptosis 4.75 (4.75 ± 0.01)] apoptosis occurred (Figure 2B). In contrast to those minor effects, when 200 μg/mL LPS was added to group C (Figure 2C), the whole image of group C showed marked clustering with ~49.12% (49.12 ± 0.01, *P* < 0.05) of cells showing early and late apoptosis. Thus, these data confirmed this dose of LP was ideal as a “mastitis model” in the follow-up experiment (Figure 2).

### Effect of TTO on Apoptosis of LPS-Induced BMEC

The blank control group A (Figure 2D, *P* < 0.05) showed apoptosis of BMEC without any treatment. The proportion of living cells was 92.10% (92.10 ± 0.03), the proportion of early apoptotic cells was 2.08% (2.08 ± 0.01) and the proportion of late apoptotic cells was 4.44% (4.44 ± 0.02). In group B, BMEC treated with 200 μg/mL LPS showed apoptosis. Among these cells, the proportion of living cells was 50.66% (50.66 ± 0.02), the proportion of early apoptotic cells was 45.70% (45.70 ± 0.01) and the proportion of late apoptotic cells was 3.42% (3.42 ± 0.01). The early withering of samples treated with 0.0002% TTO+LPS, 0.0004% TTO+LPS, 0.0006% TTO+LPS, 0.0008% TTO+LPS, 0.001% TTO+LPS, 0.002% TTO+LPS, 0.004% TTO+LPS, 0.006% TTO+LPS, 0.008% TTO+LPS, and 0.01% TTO+LPS was 61.55% (61.55 ± 0.04), 41.30%(41.30 ± 0.03, *P* < 0.05), 22.15% (22.15 ± 0.05, *P* < 0.05), 40.14% (40.14 ± 0.03, *P* < 0.05), 49.31% (49.31 ± 0.03, *P* < 0.01), 55.69% (55.69 ± 0.01, *P* < 0.01), 52.56% (52.56 ± 0.03, *P* < 0.01), 55.46% (55.46 ± 0.02, *P* < 0.01), 52.87% (52.87 ± 0.02, *P* < 0.01), and 43.67% (43.67 ± 0.02, *P* < 0.01), respectively (Figures 2F–O). After adding different concentrations of TTO (Figures 2F–O), the analysis indicated that TTO in group G (Figure 2G), H (Figure 2H), and I (Figure 2I) elicited protective effects, especially group H. The proportion of living cells, early apoptotic cells and late apoptotic cells was 71.95, 22.15, and 5.11%, respectively.

### Effect of TTO on Inflammatory and Apoptotic Factors in the LPS-Induced Mastitis Model

**Figure 3:**
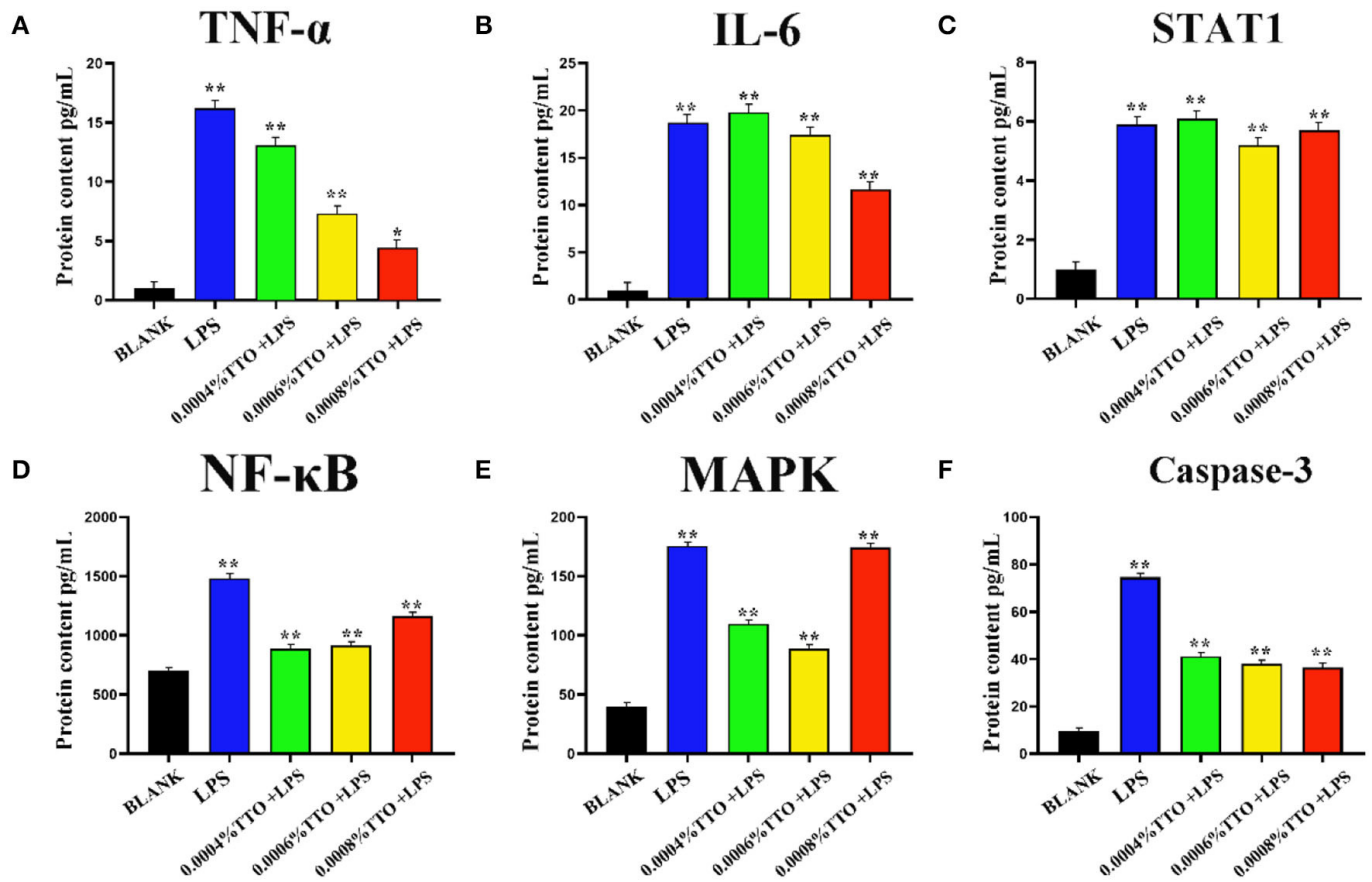
Effect of different concentrations of TTO on protein concentration associated with inflammatory response in BEMC with LPS challenge. **(A)** Protein concentrations of TNF-α (pg/ml). **(B)** Protein concentrations of IL-6; **(C)** Protein concentrations of STAT1; **(D)** Protein concentrations of NF-κB; **(E)** Protein concentrations of MAPK4; **(F)** Protein concentrations of caspase-3 Data were presented as means ± s.d. of at least three independent experiments, *P < 0.05, **P < 0.01 using two tailed student *t*-test.

Concentrations of TNF-α and IL-6 in the 200 μg/mL LPS group were more than 15-times higher than the BLANK (*P* < 0.01). Additionally, compared with the BLANK, STAT1 increased almost 6-times after addition of TTO at 0.0004% (*P* < 0.01), 0.0006% (*P* < 0.01), and 0.0008% (*P* < 0.05), respectively. Increased TTO concentrations led to decreased concentrations of TNF-α (*P* < 0.01) and IL-6 (*P* < 0.01), with a more pronounced effect on TNF-α. Expression of STAT1 increased slightly upon addition of 0.0004% TTO (*P* < 0.01). Protein concentrations of TNF-α, IL-6 and STAT1 were significantly downregulated with 0.0006% (*P* < 0.01) and 0.0008% (*P* < 0.01) TTO supplementation (Figures 3A–C). After addition of 200 μg/mL LPS, the LPS group had a significant increase in protein concentrations of NF-κB (*P* < 0.01), MAPK4 (*P* < 0.01), and caspase-3 (*P* < 0.01) (Figures 3D–F). The protein expression levels of NF-κB (*P* < 0.01), MAPK4 (*P* < 0.01), and caspase-3 (*P* < 0.01) were significantly reduced in the groups treated with TTO.

### Transcriptome Analysis

**Figure 4:**
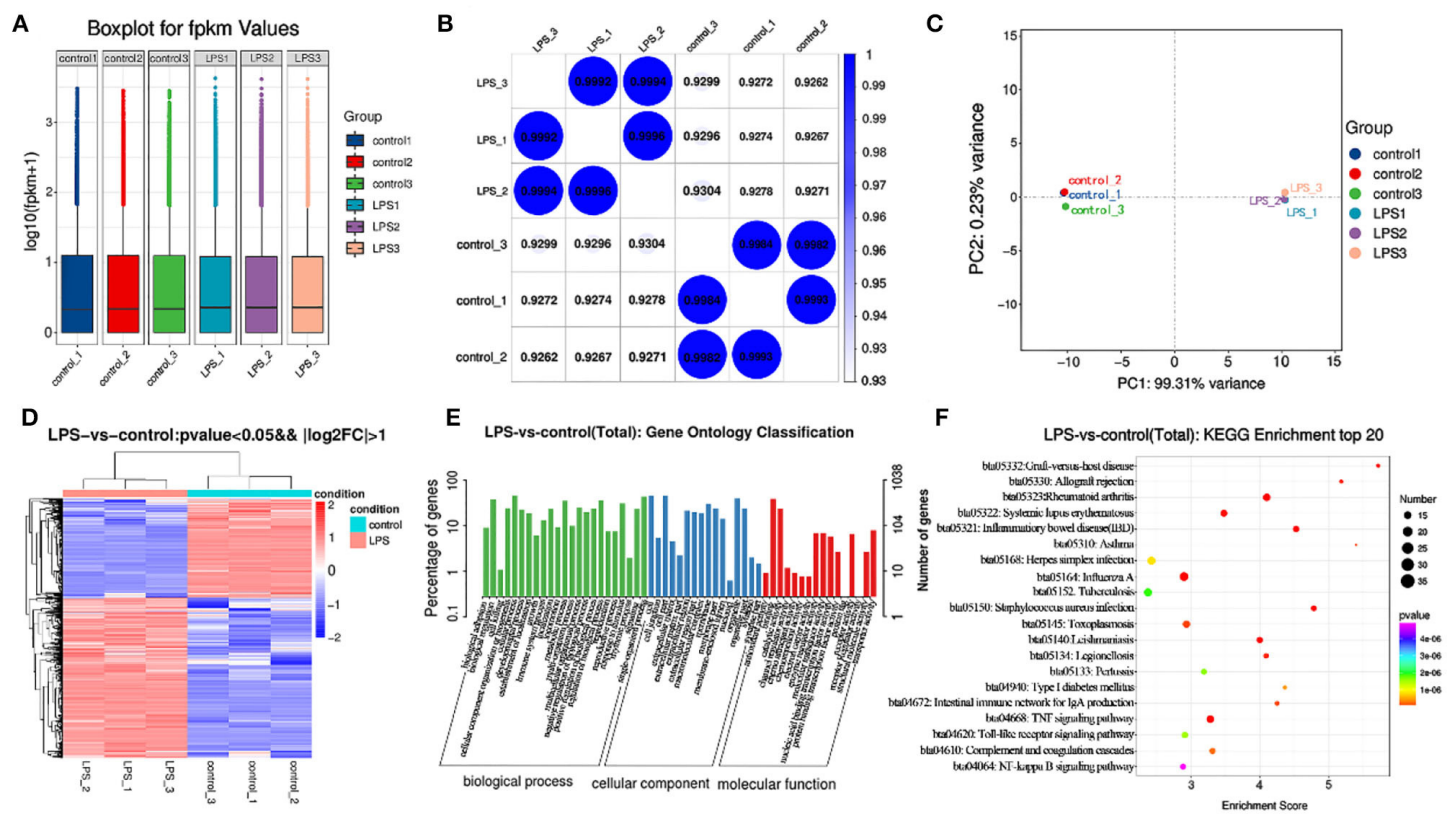
Transcriptome analysis of control and LPS group. **(A)** Box line diagram in control and LPS group. The abscissa is the sample name and the ordinate is log^10^ (fpkm + 1). **(B)** Thermal diagram of the correlation coefficient between samples. The abscissa represents the name of the sample, and the ordinate represents the name of the corresponding sample. The color represents the size of the correlation coefficient. **(C)** PCA diagram in control and LPS group. **(D)** Screening for differentially expressed genes between control and LPS-induced BMEC. **(E)** GO enrichment. Horizontal axis is the GO entry name and the vertical axis is the –log^10^ *p*-value. **(F)** KEGG enrichment, top 20 genes.

After building LPS induced mastitis model, we want to study its transcriptome level. Different genes were obtained by high-throughput sequencing analysis to provide data support for subsequent research. RNA-seq was used to sequence the LPS (200 μg/ml) induced model for 12 h. Considering the potential impact of the data error rate on the results, we used trimmatomatic software to preprocess the quality of the original data and to generate a statistical summary of the number of reads in the whole quality control process (Table S1). Fpkm is one of the most commonly-used methods to estimate expression level of protein-coding genes (Table S2). The degree of symmetry and dispersion also was deemed appropriate (Figure 4A, GEO databases: SRR11862300, SRR11862301, SRR11862299, SRR11862298, SRR11862297, SRR11862296).

The similarity of the LPS group was close to 1 (Figure 4), and that of the control was close to 1 (Figure 4B). Principal component analysis (PCA) indicated close concordance among samples in the LPS and control groups, underscoring the validity of the data generated (Figure 4C).

A total of 1270 mRNAs were identified as differentially expressed, of which 787 genes were upregulated and 483 downregulated. The differentially expressed genes included *TNF-*α, *IL6, STAT1*, and *MAPK4*. Among these genes, *TNF-*α and *IL6* were significantly upregulated. The difference multiples were 4.41 and 6.28 times, respectively (Figure 4D, Table S3).

The GO annotation results indicated that differentially expressed mRNAs participate in biological adhesion, biological regulation, cell killing, cellular component organization or biogenesis, cellular process, developmental process, growth, immune system process, negative regulation of biological process, positive regulation of biological process, and cell junction among others (Figure 4E).

Among the top 20 KEGG pathway entries, the differentially expressed mRNAs participate in TNF signaling, rheumatoid arthritis, inflammatory, *Staphylococcus aureus* infection, systemic lupus erythematosus, graft-vs-host disease, allograft rejection, intestinal immune network for IgA production, type I diabetes mellitus, herpes simplex infection, toll-like receptor signaling pathway, and NF-κB signaling pathway among others (Figure 4F).

### Physiological Gene Function Evaluation

**Figure 5:**
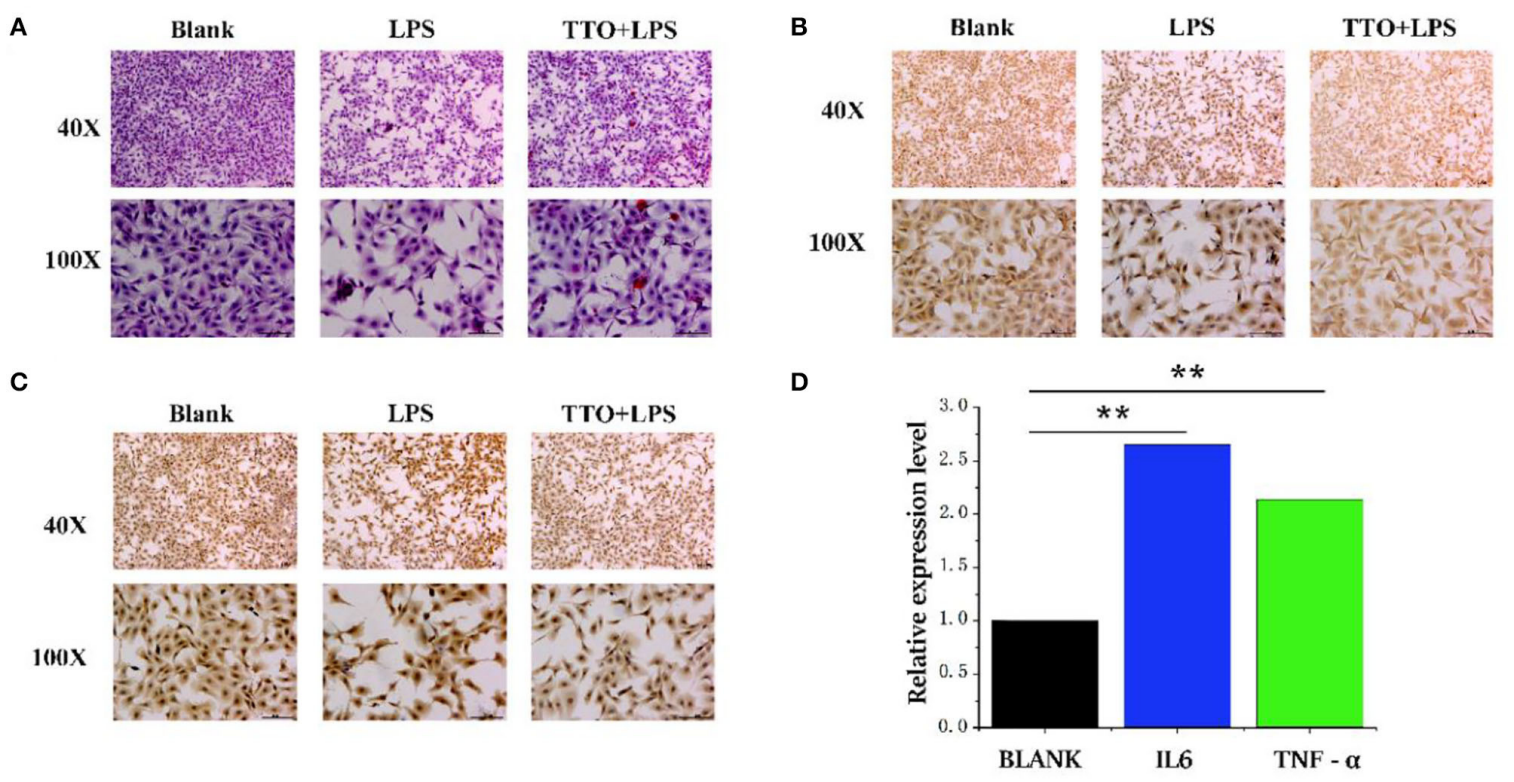
Tea tree oil induced the expression changes of TNF-α and IL 6 in BMEC. **(A)** HE staining of BMEC in LPS (200 μg/ml) and TTO (0.008%) + LPS (200 μg/ml) of 12 h; **(B)** TNF-α immunohistochemical of BMEC in LPS (200 μg/ml) and TTO (0.008%) + LPS (200 μg/ml) of 12 h; **(C)** IL-6 immunohistochemical of BMEC in LPS (200 μg/ml) and TTO (0.008%) + LPS (200 μg/ml) of 12 h. **(D)** Sequencing results of TNF-α and IL 6 expression level. Black bar represents BLANK; blue bar represents IL6; green bar represents TNF-α. Data were presented as means ± s.d. of at least three independent experiments, **P < 0.01 using two tailed student *t*-test.

Compared with BLANK, cells treated with LPS showed a heighten degree of apoptosis. However, the TTO (0.008%) + LPS (200 μg/ml) group inhibited this state (Figure 5A). Immunohistochemical results showed that cells treated with LPS also had greater protein concentrations of TNF-α and IL6. The expression of TNF-α and IL6 increased significantly in the TTO + LPS group (Figures 5B,C). The expression of *TNF-*α (*P* < 0.01) and *IL-6* (*P* < 0.01) detected by RNA-seq was consistent with immunochemical results. In addition, sequencing results also coincided with immunohistochemical data (Figure 5D).

## Discussion

LPS, a macromolecular structural component on the outer membrane of gram-negative bacteria (14, 15), can trigger an immune response in mammalian cells leading to the release of pro-inflammatory factors. Previous research underscored that the whole process of mastitis can be simulated using an LPS-induced challenge of BMEC (15, 16). In the current study, the proliferation activity of BMEC was enhanced subsequent to LPS (50 μg/mL) challenge; however, it decreased when the concentration of LPS was >100 μg/mL, which is consistent with previous studies (17). Of particular interest was the improvement of immune system activity and increased proliferation activity of cells at the low concentration of LPS; whereas, a high concentration of LPS led to a serious inflammatory reaction followed by apoptosis. These responses suggested that there is a dose-effect of LPS on regulating BMEC homeostasis. Thus, available data support the idea that LPS might play a dual role in modulating proliferation and inflammatory response in BMEC.

Tea tree oil has significant inhibitory on *E. coli* and endotoxins (18). Gustafson et al. reported that TTO can promote autolysis of *E. coli* and induce a noticeable inhibitory effect on LPS-induced inflammation (19). Thus, we speculate that TTO might play a positive role in protection against cow mastitis. In the present study, flow cytometry results showed that the proportion of normal living BMEC stimulated by LPS increased after TTO supplementation at an appropriate concentration (<50 μ g/ml LPS). Similarly, the proportion of early apoptosis, late apoptosis and dead cells decreased. Additionally, the LPS-induced inflammation was supported by the release of pro-inflammatory cytokines. It is well-established that BMEC produce TNF-α, IL-6 and STAT1 during acute inflammation induced by LPS (20). TNF-α is a major cytokine during the early stages of infection, which in *E. coli* mastitis is closely related to endotoxin shock (21). IL-6 is a pleiotropic cytokine that mediates many immune and inflammatory reactions (22). Our results showed that TTO could attenuate the expression of TNF-α and IL-6 induced by LPS, with a more pronounced suppression of TNF-α. STAT1 promotes apoptosis, inhibits cell growth and differentiation, and plays an important role in inhibiting the occurrence and development of tumors. Overall, our results suggest that supplementation of TTO might help alleviate inflammation at least partly due to downregulated pro-inflammatory cytokines caused by high concentrations of LPS.

Previous studies have shown that inflammatory cytokines are primarily produced by activation of the NF-κB and MAPK signaling pathways, while apoptosis-promoting factors are mainly produced by activation of the caspase-3 pathway (23, 24). To further explore the mechanism of TTO inhibition the production of inflammatory cytokines and pro-apoptotic factors, we measured protein concentrations of NF-κB, MAPK4 and caspase-3 in response to TTO. NF-κB, MAPK4, and caspase-3 were greater in LPS-infected BMEC and decreased significantly after addition of TTO, suggesting that an appropriate concentration of TTO inhibits the production of NF-κB, MAPK4, and caspase-3. Therefore, we speculate that TTO might alleviate inflammatory responses in BMEC via NF-κB, MAPK4, and caspase-3 signaling pathways. The previous study sequenced the transcriptome of BMEC infected by *Staphylococcus aureus, E. coli* and *Klebsiella pneumoniae* using the Solexa system, and GO analysis indicated that the differentially expressed genes in the infected and non-infected groups were enriched in cell metabolism, apoptosis and embryonic development (25). Additionally, cluster analysis of homologous proteins revealed that they participate in translation, ribosome biosynthesis and repair. Oxidative phosphorylation pathway, nod-like receptor pathway and apoptosis pathway were identified as three enriched pathways via KEGG analysis.

The acute clinical indicators caused by LPS are closely related to the enzyme activities and acute-phase proteins in milk from cows with mastitis caused by *E. coli*. LPS stimulation resulted in rapid immune response in BMEC with the most active cellular response detected at 4 h. The most active immune response pathway included the RIG-I-like receptor signaling pathway, nod like receptor signaling pathway and MAPK signaling pathway. Wang et al. sequenced the transcriptome of mammary gland infected with S56, S178, and S36 *Staphylococcus aureus* strains and screened 1720, 427, and 219 differentially expressed genes, respectively (26). GO and pathway analysis in this research showed that these genes are involved in the inflammatory response, metabolic transformation, cell proliferation and apoptosis signaling pathways. Our research showed that Interleukin1 α (IL-1α), TNF, homo sapiens ephrin-B1, IL-8, and early growth response 1 were upregulated. These data provided a reference for mastitis-related gene transcription, post-transcriptional regulation, and the host cell immune response to pathogens. Findings were consistent with the differentially expressed genes determined in this study. Overall, new genes uncovered in the present study might be potentially used as biomarkers for diagnosis and prevention of clinical mastitis in dairy cows. In addition, our preliminary identification of gene functions may help elucidate the molecular mechanism of LPS-induced mastitis at the gene network.

## Data Availability Statement

The datasets generated for this study can be found in GEO, Accession No.'s SRR11862300, SRR11862301, SRR11862299, SRR11862298, SRR11862297, SRR11862296.

## Ethics Statement

The animal use protocol was approved by the Institutional Animal Care and Use Committee in the College of Animal Science and Technology, Yang Zhou University, Yang Zhou, China.

## Author's Note

This manuscript has been released as a pre-print at Research Square, https://www.researchsquare.com/article/rs-18655/v1 (ZC, YZ, JZ, et al.).

## Author Contributions

ZC and ZY conceived and designed the experiments. ZC, JZ, YZ, and LL performed the experiments. ZC, XW, YL, JL, DG, HX, and ZY analyzed the data. ZC, JL, YL, and DG wrote the paper. All authors contributed to the article and approved the submitted version.

## Conflict of Interest

The authors declare that the research was conducted in the absence of any commercial or financial relationships that could be construed as a potential conflict of interest.

## Supplementary Material

Table S1 Screening for mRNAs with differential expression in the LPS-induced BMECs.

Table S2 Pretreatment results of sequencing data quality.

Table S3 Distribution statistics of fpkm value of genes.
